# CB2 Receptor Involvement in the Treatment of Substance Use Disorders

**DOI:** 10.3390/biom11111556

**Published:** 2021-10-20

**Authors:** Francisco Navarrete, María S. García-Gutiérrez, Ani Gasparyan, Daniela Navarro, Jorge Manzanares

**Affiliations:** 1 Instituto de Neurociencias, Universidad Miguel Hernández-CSIC, Avda. de Ramón y Cajal s/n, San Juan de Alicante, 03550 Alicante, Spain; fnavarrete@umh.es (F.N.); maria.ggutierrez@umh.es (M.S.G.-G.); agasparyan@umh.es (A.G.); dnavarro@umh.es (D.N.); 2 Red Temática de Investigación Cooperativa en Salud (RETICS), Red de Trastornos Adictivos, Instituto de Salud Carlos III, MICINN and FEDER, 28029 Madrid, Spain

**Keywords:** cannabinoid 2 receptor, substance use disorder, reward system, alcohol, cocaine, nicotine

## Abstract

The pharmacological modulation of the cannabinoid receptor 2 (CB2r) has emerged as a promising potential therapeutic option in addiction. The purpose of this review was to determine the functional involvement of CB2r in the effects produced by drugs of abuse at the central nervous system (CNS) level by assessing evidence from preclinical and clinical studies. In rodents, several reports suggest the functional involvement of CB2r in the effects produced by drugs of abuse such as alcohol, cocaine, or nicotine. In addition, the discovery of CB2r in brain areas that are part of the reward system supports the relevance of CB2r in the field of addiction. Interestingly, animal studies support that the CB2r regulates anxiety and depression behavioral traits. Due to its frequent comorbidity with neuropsychiatric disorders, these pharmacological actions may be of great interest in managing SUD. Preliminary clinical trials are focused on exploring the therapeutic potential of modulating CB2r in treating addictive disorders. These promising results support the development of new pharmacological tools regulating the CB2r that may help to increase the therapeutic success in the management of SUD.

## 1. Introduction

Substance use disorder (SUD) is a chronic and relapsing mental illness characterized by compulsive drug seeking and the repeated achievement of episodes of intoxication and withdrawal. It is a clinical entity diagnosed according to the criteria of the fifth edition of the Diagnostic and Statistical Manual of Mental Disorders (DSM-V) [1]. Such measures enter into the following subgroups: (1) alterations in the control of substance use, (2) impairment of the social component, (3) use under risk conditions, and (4) development of tolerance. These may include the lack of control of substance use occurring often or more extended periods than initially planned. These alterations may also have an intense desire and a high degree of impulsivity, which translate into an almost exclusive dedication to the search, consumption, and recovery of the effects induced by the substance.

According to the latest report issued by the United Nations Office on Drugs and Crime (UNODC), approximately 275 million people worldwide consumed some drug of abuse at least once during 2019 [2]. Thirty-one million people who use drugs meet diagnostic criteria for SUD and are candidates for treatment. According to the World Health Organization (WHO) data, deaths directly related to SUD amounted to 167,750 in 2015, representing a 60% increase over the previous figure of 2000. Opioids produce the highest morbidity and mortality among the drugs consumed, accounting for 76% of the deaths mentioned above (mainly due to overdose). On the other hand, alcohol constitutes the most consumed legal drug, globally ranking seventh as a cause of death or loss of quality of life [3]. Likewise, cannabis is the illegal drug with the highest percentage of users, amounting to 192 million during 2016 [2].

One of the most significant difficulties accompanying the therapeutic approach to SUD is the low rate of people who, meeting diagnostic criteria, finally receive adequate treatment, currently estimated as seven in eight people [2]. A worrying fact is the lack of suitable drugs to reach complete dishabituation or significant reduction in consumption, minimizing the associated risks. Proof of this is the absence of approved treatments for withdrawal from cannabis, cocaine, or amphetamine derivatives. In these cases, symptomatic approaches may reduce characteristic components after cessation of use, such as anxiety or irritability. Even for drugs officially approved to treat alcohol, tobacco, or opiate dependence by the leading regulatory agencies Food and Drug Administration (FDA) in the United States, or the European Medicines Agency (EMA) in Europe, the percentage of patients recovering entirely or partially is significantly reduced. In this sense, it is essential to improve our knowledge of the neurobiological bases underlying the addictive process for all these reasons. In this regard, it is crucial to identify the mechanisms regulating the reward system in the mesolimbic/mesocortical pathway (from the ventral tegmental area (VTA) to the nucleus accumbens (NAcc) and or the prefrontal cortex (PFC)). Information on new targets that the consumption of different abuse substances may modify is critical for developing pharmacological strategies.

### The Endocannabinoid System: Role in SUD

ECS is a ubiquitous lipid signaling system distributed throughout the organism that participates in multiple intracellular signaling pathways [4,5]. Cannabinoid receptors, endogenous ligands or endocannabinoids (eCBs), and their synthesizing and degrading enzymes are the main components of the ECS, present in the central and peripheral nervous system [6,7], and in many other peripheral tissues regulating distinct functions [8].

The CB1 cannabinoid receptor (CB1r) is the most abundant G protein-coupled receptor in the brain [9]. Physiological actions of endocannabinoids in the CNS are mediated by the activation of CB1r [10]. Its expression in the CNS is widespread and heterogeneous and have crucial roles regulating brain function and disease processes [11,12,13]. The CB2 cannabinoid receptor (CB2r) was initially considered as a peripheral cannabinoid receptor due to its high expression in the rat spleen [14] and leukocyte subpopulation in humans [15], participating in the regulation of the immune system [16]. The first findings identified the presence of CB2r in the CNS only under pathological conditions such as in senile plaques in Alzheimer’s disease [17], activated microglial cells/macrophages in multiple sclerosis, spinal cord in amyotrophic lateral sclerosis [18] and in the vicinity of tumors [19]. However, Van Sickle and colleagues revealed that CB2r is expressed in neurons of the brainstem of mice, rats and ferrets under normal conditions [20]. This finding significantly increased the interest of CB2r in the regulation of brain function [21,22,23,24,25]. Interestingly, CB2r was detected not only in microglia [26] but also in neurons [25,27,28] and astrocytes [29].

The eCBs are lipid messengers acting as in paracrine, autocrine and probably endocrine mode, because their lipid nature allows them to diffuse and cross membranes [4,5,6,30,31]. eCBs are agonists of CB1r and CB2r that are not accumulated in secretory vesicles but rather synthesized under tonic or phasic (on demand) modes, and released to the extracellular space following physiological and pathological stimuli [32]. The two main eCBs are derivatives of polyunsaturated fatty acids, N-arachidonoylethanolamine (anandamide, AEA) [33] and 2-arachidonoylglycerol (2-AG), being the most abundant eCBs in the brain [34].

The ECS has received particular attention in recent years [35]. Numerous publications initially focused on the therapeutic potential of CB1r [36,37,38,39]. Several authors showed how the blockade of this receptor in animal models could regulate the reinforcing and motivational properties of alcohol [40,41,42,43]. Indeed, clinical trials were conducted to test its potential therapeutic utility [44,45], but the lack of efficacy in patients and the occurrence of specific adverse severe effects forced the abandonment of this strategy. Among the alternatives that were sought, pharmacological modulation of the CB2r emerged as a promising option in the field of addiction following the results obtained so far [46,47,48,49,50,51]. The activation of the CB2r does not induce the addictive properties of the CB1r (reinforcing effects, cognitive alterations), exhibits anti-inflammatory [52,53] and neuroprotective potential [54,55]. Furthermore, the modulation of CB2r induces anxiolytic [56,57] and antidepressant activity [24,58] that may be of great interest in the management of SUD because of its frequent comorbidity with other neuropsychiatric disorders such as anxiety, depression, and psychosis.

## 2. Methods

The literature review consisted of a search for scientific information in the Medline database (PubMed) employing Medical Subject Headings (MeSH). A total of three search boxes were employed according to the total of drugs included in the review: alcohol (“Ethanol” [MeSH]), cocaine [MeSH], and nicotine [MeSH]. These terms were combined with “Receptor, Cannabinoid, CB2” [MeSH] by the Boolean operator “AND”. All the results for each search were critically analyzed by all the authors to decide the selection of each reference according to the adequacy of its content with the subject matter of the study. No PubMed filters were applied to maximize the selection of all the available and appropriate information. All original articles, systematic reviews or meta-analyses focusing on the role of CB2r on drug addiction were accepted. Those articles not related to the topic of interest, not written in English or to which access was not possible were discarded.

## 3. Expression, Distribution, and Functional Involvement of CB2r in the Reward System

Since the publication of van Sickle et al. [20] describing for the first time the presence of CB2r in brainstem neurons of mice, rats, and ferrets under normal conditions, numerous studies have been carried out to determine its expression, distribution and functional involvement in different brain areas. George Uhl’s group was the first to describe the distribution of CB2r in different brain areas of the rat brain using immunohistochemical techniques [21]. Subsequently, this same group analyzed the gene expression of CNR2 isoform A (brain-tissue-specific) in human NAcc, cerebral cortex (CCx), amygdala (Amy), and mouse striatum and PFC [59]. One year later, our group demonstrated that under normal conditions, the gene encoding for CB2r is expressed in different mouse brain areas related to the regulation of emotional response (Amy, hippocampus, and raphe nuclei) or reward (NAcc and VTA) [24]. Among the findings obtained shortly after that, the discovery of CB2r in regions related to the reward system was of great interest, suggesting its possible role in regulating addictive processes. These results suggested the involvement of CB2r in the regulation of various functional aspects at the brain level, highlighting the possible modulation of the reinforcing and motivational effects of drugs of abuse.

**Figure 1:**
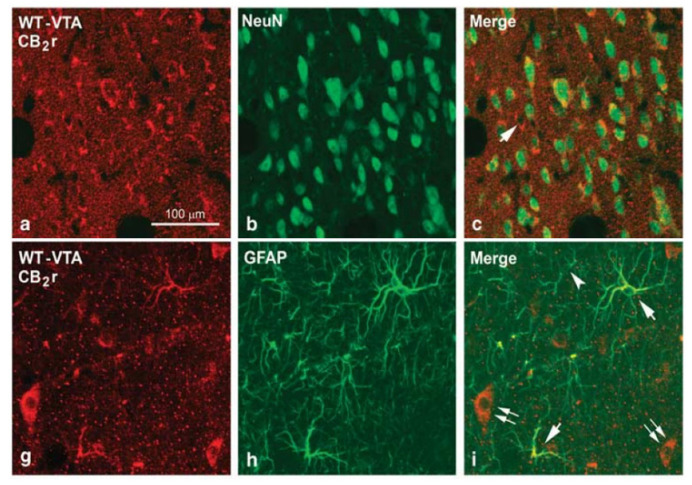
Confocal photomicrographs showing immunolabeling for CB2 receptors (CB2r), neuronal nuclei (NeuN) and glial fibrillary acidic protein (GFAP) in the ventral tegmental area (VTA) and the nucleus accumbens (NAcc) of wild-type (WT) mice. Double labeling CB2r-NeuN (yellow cells in c) in the VTA and the NAcc and CB2r-GFAP (yellow cells in i) in the VTA indicates the existence of CB2r in neurons and astrocytes. Image adapted from Aracil-Fernandez et al. Neuropsychopharmacology (2012) 37, 1749–1763.

Subsequently, Xi et al. [60] provided information on the regulation of dopaminergic neurotransmission in the NAcc mediated by CB2r. Both systemic and intracerebral (NAcc) administration of the compound JWH133 (3, 10 and 20 mg/kg, intraperitoneal (i.p.) administration; 1, 10, 100 and 1000 M, intraNAcc administration), a selective and potent CB2r agonist, attenuated the increase in dopamine concentrations in the NAcc induced by cocaine self-administration in mice. The authors proposed two hypotheses that may explain this effect. First, there was a reduced basal expression of CB2r in mesolimbic dopaminergic neurons of the VTA, whose activation would produce an inhibitory effect reducing dopamine release in the NAcc. Second, activation of CB2r located in microglia or astrocytes of the VTA or the NAcc could indirectly inhibit dopamine release in the NAcc due to the release of cytokines and inflammatory factors. In this regard, further work has shown that CB2r is expressed in both neurons and astrocytes in the VTA and NAcc of ICR wild-type (WT) mice (Figure 1) [48]. Furthermore, this same study revealed that CB2r colocalizes with dopaminergic receptor 2 (D2R) in neurons, suggesting that CB2r might functionally cooperate with the mesolimbic dopaminergic system. Indeed, studies by Zhang et al., employing a multidisciplinary approach first in mice [25] and then in rats [61], confirmed that CB2r is present in VTA dopaminergic neurons projecting to the NAcc. Functionally, activation of CB2r by administration of the agonist JWH133 under ex vivo and in vivo conditions (10 and 20 mg/kg, i.p.; 1 and 3 µg/side, intraVTA administration; 1 and 10 M, in vitro experiments with brain slices containing the VTA), reduced the firing rate and dopamine release in VTA dopaminergic neurons. Furthermore, local administration of JWH133 (1 and 3 µg/side) in the VTA significantly reduced cocaine self-administration. More recently, it was suggested that CB2r could modulate the excitability of VTA dopaminergic neurons through both synaptic and intrinsic mechanisms, involving the reduction of presynaptic glutamate release as well as the enhancement of postsynaptic neuronal M-type K^+^ currents [62]. These results confirm the role of the CB2r on the regulation of dopaminergic neurotransmission and suggest this receptor as a new therapeutic target for SUD management.

## 4. Therapeutic Potential of CB2r in the Management of Substance Use Disorders

The information mentioned so far suggests that CB2r may present a relevant role in the functional regulation of the reward system. Evidence accounting for its involvement in the regulation of the reinforcing actions of substances such as alcohol, cocaine, or nicotine is detailed in this section. In addition, CB2r participates in other mechanisms closely related to the addictive process. For instance, this receptor is involved in the regulation of cognitive processes [63,64], the control of impulsive behavior [65], aggressiveness [66] or emotional responsiveness [24,56,57,67].

### 4.1. CB2r and Alcohol

The first reference related to the involvement of CB2r in addiction, and more specifically with the effects of alcohol, was described by Emmanuel Onaivi’s group [51,68]. Using a model of voluntary alcohol consumption (also called two-bottle choice test, one containing water and the other a defined concentration of alcohol that is progressively increased), it was observed that mice that consumed a higher amount of alcohol were characterized by a lower gene expression of CNR2 at the mesencephalic level. Furthermore, the authors evaluated the effect of systemic administration of the CB2r agonist (JWH015, 20 mg/kg, i.p.) on alcohol consumption in mice previously exposed to a chronic mild stress model (CMS). A marked increase in alcohol consumption was observed in stressed mice with no change in unstressed mice. The effects of the CB2r antagonist (AM630, 3 mg/kg, i.p.) were also analyzed without finding differences in either of the two groups evaluated. This same study describes for the first time the association between the CNR2 polymorphism, Q63R, and alcoholism in a population of Japanese patients. This polymorphism would be related with a reduced CB2r-mediated response and, consequently, a greater vulnerability for the development of alcoholism.

Subsequent studies reinforced the relationship between lower CB2r expression and higher alcohol consumption. In a model of continuous (liquid diet with 10% alcohol, 15 days) or intermittent (liquid diet with 10% alcohol, 5 days/week, 3 weeks) alcohol access, the effects of alcohol withdrawal, after 6 or 24 h, on CNR2 gene expression in the Amy of male Wistar rats were evaluated, showing a reduction that was more emphasized at 24 h [69]. Similarly, intermittent alcohol exposure (ethanol (EtOH) 20% *w*/*v*, 3 g/kg, i.p., 4 days/week, 4 weeks) of male Wistar rats produced a marked reduction of CNR2 gene expression in the striatum and hippocampus, which was assessed 2 weeks after the end of the procedure [70]. In a complementary manner, post-mortem studies analyzed CNR2 gene expression in the dorsolateral PFC and NAcc of patients with alcohol dependence criteria [71], revealing a reduction in both regions. These changes could be attributed to prolonged consumption and the greater vulnerability of these patients to the reinforcing effects of alcohol. Another approach used to investigate the role of CB2r in regulating the reinforcing properties of alcohol is the use of genetically modified animals. In this regard, the study performed with Swiss ICR mice devoid of the CNR2 gene in the central nervous system, CB2-/-, shows how the absence of CB2r is related to a greater preference for the conditioned compartment in the conditioning place preference (CPP) test, greater preference, and alcohol consumption in the two-bottle system, and greater motivation and alcohol consumption in the oral ethanol self-administration test [50]. Moreover, the results obtained in the self-administration paradigm were replicated in other CB2-/- mice in the central nervous system but based on C57BL/6J, a strain characterized by a high vulnerability for alcohol consumption. Therefore, these results revealed that genetic deletion of CNR2 significantly increased the vulnerability of the animals to the reinforcing and motivational stimuli of alcohol.

These findings may not be explained by possible alterations in the peripheral metabolization of ethanol, as no differences were found in plasma ethanol concentrations, after administration at different doses, between the CB2-/- mice and their corresponding control group. On the other hand, gene expression analyses showed that basally CB2-/- mice presented a higher expression of the µ-opioid receptor (MOr) in the NAcc than their control group. In addition, after administration of different doses of ethanol, a marked increase of tyrosine hydroxylase (TH) gene expression in the VTA and MOr in the NAcc was observed. These data indicate that these alterations may be related, at least in part, to the increased alcohol consumption and motivation of CB2-/- mice.

Subsequently, forced (one bottle with EtOH 16% *v*/*v*, 5 months), intermittent (one bottle with EtOH 16% *v*/*v*, 4 days/week, 5 months), and voluntary (two bottles with water or EtOH 8% *v*/*v*, 3 months) drinking models were employed in CB2-/- mice under both group housing and isolation conditions [72]. The results showed no differences in the forced consumption paradigm considering both genotype and housing conditions. In contrast, in the intermittent drinking model, a significant increase in alcohol consumption was observed in group-stabled CB2-/- mice, with no differences found in isolated mice. These results indicate that the absence of CB2r would be responsible for increased alcohol consumption despite group housing, a condition that is usually associated with a lower consumption rate compared to isolation. This result is consistent with previous studies that highlighted the involvement of CB2r in the regulation of the stress response [57,67]. Finally, contrary to the previously discussed results [50], in the voluntary consumption paradigm, a significant reduction in the level of consumption and preference (10–12 weeks) was found in CB2-/- mice. The causes that may explain finding an opposite alcohol consumption profile between the two studies are unknown. Considering that they employ the same mouse strain and genotype, possible differences in the performance of the experimental procedures or the mice’s housing conditions could be responsible for these discrepancies.

**Figure 2:**
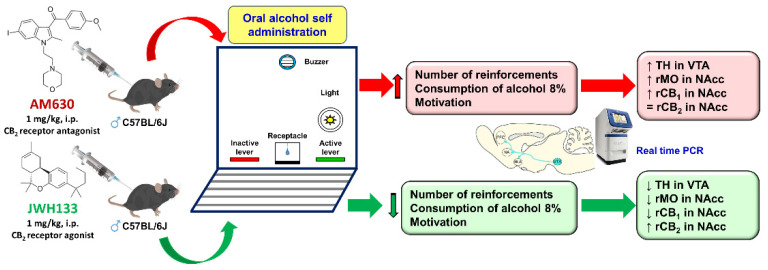
Representative picture of the experimental procedure and the main findings obtained with pharmacological modulation of CB2 receptors (CB2r) in the oral ethanol self-administration paradigm. Image adapted from Navarrete et al. Biochemical Pharmacology (2018) 157, 227–234.

Apart from CB2-/- animals, pharmacological studies were performed with compounds that selectively activated or blocked CB2r. Thus, Powers et al. [73] again employed CB2-/- mice, replicating their increased degree of conditioning by the environment in which they receive alcohol (20%, 2 g/kg, i.p.). They also assessed the effects of administration of drugs with agonist (JWH133, 10 and 20 mg/kg) or antagonist (AM630, 10 and 20 mg/kg) action on CB2r in WT mice (HS/Ibg strain). Pharmacologically, the authors found no effect on alcohol consumption or place preference conditioning. This finding suggested that neurodevelopmental modifications in CB2-/- mice were likely behind the changes in vulnerability to the reinforcing effects of alcohol. However, Al Mansouri et al. [74] demonstrated that administration of β-caryophyllene, a selective CB2r agonist, significantly reduced voluntary alcohol consumption and conditioned place preference in males C57BL/6J strain mice, effects that were blocked by the antagonist AM630. Similarly, Liu et al. [28] also showed that administration of JWH133 (5 mg/kg, i.p.) completely blocked place preference induced by alcohol administration. Recently, it has been shown how pharmacological modulation of CB2r by the antagonist AM630 (1 mg/kg, i.p.) significantly increased the number of lever activations, as well as the level of alcohol consumption and motivation in the oral self-administration experimental paradigm [75]. These findings agree with the profile observed in CB2-/- mice [50] and are related to reducing CNR2 expression levels upon alcohol exposure in different experimental conditions [69,70]. In this same study, it is shown how CB2r activation mediated by the agonist JWH133 (1 mg/kg, i.p.) produces the opposite effects, significantly reducing alcohol self-administration, a result consistent with those obtained previously [28,74], as well as more recently in the CPP paradigm where CB2r activation reduced alcohol-rewarding behaviors [76]. It should be noted that these changes were accompanied by bidirectional modifications in the gene expression of brain targets intimately related to the regulation of the reinforcing effects of alcohol such as TH in the VTA, and MOr and CNR1 and CNR2 in the NAcc (Figure 2). Discrepancies regarding the effects of pharmacological regulation of CB2r on alcohol consumption and motivation may be due to various experimental circumstances. We may use different mouse strains (HS/Ibg or C57BL/6J) or different procedures to assess alcohol consumption (two-bottle system, place preference conditioning, or oral self-administration). Additionally, pharmacological aspects such as the dose, route, or administration schedule could play an important role. In short, further studies exploring new experimental conditions and contributing to elucidate the neurobiological mechanisms involved are needed.

A conditional knockout mouse was recently generated in which CNR2 expression was explicitly knocked down in DAT-Cnr2-/- dopaminergic neurons [77]. This was achieved using a recombinant expression system (Cre-LoxP) that utilized the dopamine transporter (DAT) promoter, producing DAT-Cnr2-/- mice. This approach is of great interest to elucidate the specific role of CB2r in dopaminergic neurons. DAT-Cnr2-/- mice show a significant elevation of motor activity, depressive-like behavior, or a lower level of anxiety. However, they are more vulnerable to the reinforcing effects of cocaine (place preference conditioning). Relative to the reinforcing effects of drugs such as alcohol or cocaine, DAT-Cnr2-/- mice develop less alcohol-induced place preference conditioning and exhibit a lower preference for the alcohol-containing bottle in a voluntary drinking paradigm (two-bottle system). Another finding of great interest is the altered interaction with environmental factors such as stress exposure. DAT-Cnr2-/- mice do not show a different alcohol consumption after exposure to the chronic mild stress model for 7 weeks, contrary to WT mice. Therefore, it could be hypothesized that the absence of CB2r would alter the processes of emotional response regulation [57,67], interfering with the increased vulnerability for alcohol consumption that usually occurs upon exposure to stressful stimuli.

Finally, it is essential to highlight that CB2r also seems to be involved in protective processes against the adverse effects of chronic alcohol consumption at hepatic and central levels. In 2011, it was first described that CB2-/- mice were more vulnerable to the development of steatosis and fibrinogenesis in the liver [78]. It was later proposed that pharmacological activation of CB2r, expressed in liver macrophages (Kupffer cells), protected against alcohol-induced steatosis by inhibiting various inflammatory processes [79]. At the brain level, only one study specifically explored the role of CB2r in the reduction of neuroplasticity and neurogenesis processes induced by alcohol, suggesting that CB2r activation increases the number of neurons in the subgranular zone of the hippocampus and the subventricular zone of mice chronically exposed to alcohol [80]. Therefore, such effects would further substantiate the potential therapeutic utility of pharmacological manipulation of CB2r in alcohol use disorder (AUD) (Table 1).

### 4.2. CB2r and Cocaine

Several studies highlight the functional importance of CB2r in the actions produced by psychostimulant drugs such as cocaine. Genetically modified mice overexpressing CB2r in the central nervous system (CB2xP) were exposed to different experimental paradigms to analyze the motor (motor sensitization), reinforcing (place preference conditioning), and motivational (intravenous self-administration) effects of cocaine. The results indicated that overexpression of CB2r significantly reduced motor sensitization, CPP, and the number of cocaine infusions. Furthermore, these behavioral alterations were associated with increased TH and DAT gene expression in the VTA of CB2xP mice compared to their controls. These differences could be related, at least in part, to the lower vulnerability of CB2xP mice for the motor, reinforcing, and motivational effects of cocaine. However, these effects were not related to the possible regulation of dopamine release in the NAcc. The microdialysis studies showed an increase in dopamine levels following cocaine administration in both genotypes [48].

The intravenous cocaine self-administration model analyzed the effects of the CB2r agonist JWH133 (10 and 20 mg/kg, i.p.) in WT mice and knockout mice lacking the gene coding for CB1r (CB1-/-) and CB2r (CB2-/-). Activation of CB2r by JWH133 significantly reduced the number of cocaine injections in WT mice. The fact that this effect was not modified by simultaneous administration of a CB1r antagonist (AM251, 3 mg/kg, i.p.), nor was it observed in CB1-/- mice, allowed us to rule out the involvement of CB1r. In contrast, the effects of JWH133 were blocked with the CB2r antagonist AM630 and were not observed in CB2-/- mice, confirming the involvement of CB2r. Likewise, using another CB2r agonist (GW405833, 3 and 10 mg/kg, i.p.), it was possible to replicate the results obtained with JWH133 injection. Regarding cocaine-induced motor hyperactivation, it is also noteworthy that in WT and CB1-/- mice, a significant reduction in distance traveled was observed in those mice administered JWH133 (20 mg/kg, i.p.), whereas no differences were obtained in CB2-/- mice [60].

In a complementary manner, the effects of the CB2 antagonist SR144528 (0.1, 0.3, and 1 mg/kg, i.p.) were evaluated in the intravenous cocaine self-administration test. No change was observed in the number of cocaine injections and no change in relapse induced by the presentation of a conditioned stimulus. However, when the cocaine-triggered relapse process (10 mg/kg, i.p.) was analyzed after an extinction period (10 days), CB2r blockade did produce a significant reduction [81]. Likewise, a subsequent study described how pharmacological activation of CB2r by administration of the agonist O-1966 (1, 3, 5, 10 and 20 mg/kg, i.p.) blocked cocaine-induced conditioning place preference (10 mg/kg, i.p.) [82]. These findings suggest that overexpression or pharmacological activation of CB2r can reduce the stimulant, reinforcing, and motivational properties of cocaine in the different animal models employed in mice. Significantly, these effects may be related to a reduction in dopamine release in the NAcc, a phenomenon that occurs when JWH133 (20 mg/kg, i.p.) is administered both under basal conditions and after cocaine administration. This effect appears to be specific to CB2r as the antagonist AM630 successfully blocks it and does not occur in CB2-/- mice, but it is present in CB1-/- mice [60].

Subsequently, it was shown that administration of the agonist JWH133 (10 and 20 mg/kg, i.p.) significantly reduced the number of cocaine infusions in male C57BL/6J mice as the animals’ motivation to obtain the drug. However, in male Long–Evans rats, pharmacological activation of CB2r did not change the number of injections and even significantly increased motivation. This finding could be due to differences in gene structure, mRNA splicing mechanisms, or even the protein’s structure and amino acid composition that makes up CB2r between mice and rats. In addition to the two known isoforms of the gene coding for CB2r (CNR2a and CNR2b), two additional rat-specific isoforms (CNR2c and CNR2d) were found. Differences in the gene expression profile of CB2r (isoforms A and B) were also identified in the striatum of mice and rats, a fact that explained, at least in part, the differential effects found in the self-administration paradigm [83].

In order to further explore the implication of CB2r in the regulation of cocaine effects, changes in its gene expression were evaluated following different administration patterns. In a recent study, specific times during the adolescent stage of male Sprague–Dawley rats were selected, in which cocaine (15 mg/kg, i.p.) was administered for 7 days. Analysis of CB2r protein expression in the PFC showed a significant reduction in expression only in those rats treated between days 33 and 39 from birth. However, such alteration does not persist in the adult stage, although it probably produces functional alterations that may reflect the response by cocaine consumption with possible implications in the development of the addictive process [84]. On the other hand, Bystrowska et al. [85] used male Wistar rats to evaluate the changes induced by cocaine after two types of experimental designs: (1) intravenous self-administration for 14 days, and (2) intravenous self-administration for 14 days followed by 10 days of quenching. Immunohistochemical techniques in the brain samples analyzed the levels of CB2r. The results revealed a significant decrease in CB2r levels in areas of the mesolimbic system, such as the NAcc and the PFC, in rats exposed to the extinction period after the self-administration procedure.

Finally, DAT-Cnr2-/- mice lacking CB2r in dopaminergic neurons were recently used to evaluate the effects of different psychostimulant drugs such as cocaine [86]. In DAT-Cnr2-/- mice, acute cocaine administration increased motor hyperactivation. On the other hand, in a motor sensitization model, DAT-Cnr2-/- mice increased sensitivity to the stimulatory effects of cocaine compared to WT mice. This effect occurred when the lowest dose of cocaine (10 mg/kg, i.p.) was administered, whereas the result was the opposite when the highest dose (20 mg/kg, i.p.) was used. Although the authors do not discuss the possible mechanisms underlying these differences, the deletion of CB2r in dopaminergic neurons could likely induce functional alterations not yet elucidated that modify the sensitivity to the psychomotor effects of high doses of cocaine. In the same study, we analyzed the reinforcing effects in the CPP paradigm, finding a more extended time spent in the cocaine-conditioned compartment (7.5 mg/kg, i.p., 4 days) of DAT-Cnr2-/- mice. Furthermore, the JWH133 agonist (3 mg/kg, i.p., 4 days) and cocaine (5 mg/kg, i.p., 4 days) were co-administered during the conditioning phase in WT mice, and the characteristic reinforcing effect was blocked.

The results available so far suggest that the absence of CB2r may be associated with increased vulnerability to the stimulant and reinforcing effects of cocaine. In contrast, overexpression or pharmacological activation of CB2r may benefit significantly reducing the actions induced by cocaine (Table 2). However, further studies are needed to understand better the neurobiological mechanisms underlying these effects and expand the therapeutic potential that functional modulation of CB2r would have in treating cocaine addiction.

### 4.3. CB2r and Nicotine

In recent years, there has been growing interest in exploring the involvement of the ECS in nicotine dependence. More specifically, several research groups focused on clarifying the possible role of CB2r in the acquisition of nicotine dependence and the processes of abstinence and relapse. The first approach to investigate the involvement of both CB1r and CB2r in the reinforcing effects of nicotine was published in 2006 [87]. This study employed an intravenous self-administration model in male Wistar rats, with which animals were trained to discriminate between self-administration of saline or nicotine (0.025–0.4 mg/kg). Once the animals acquired the learning criteria, the effect of administration of different drugs acting on nicotinic acetylcholine receptors and cannabinoid receptors was evaluated. The authors observed that administration of CB1r (rimonabant, 10 mg/kg, i.p.) and CB2r (SR144528, 3 mg/kg, i.p.) antagonist drugs produced no effect on the discrimination task, which initially suggested that cannabinoid receptors were not involved in modulating the reinforcing actions of nicotine.

Subsequently, Gamaleddin et al. [88] conducted a study in male Long–Evans rats to reassess the involvement of cannabinoid receptors in nicotine-induced reward effects focusing on the motivation to obtain the drug, as well as its pursuit after a period of abstinence. They performed an intravenous self-administration of nicotine to achieve the stated objectives by evaluating the effects of administering a nonselective CB1r and CB2r agonist, WIN55,212-2 (0.1, 0.3, and 1 mg/kg, i.p.), on the number of infusions. Agonist administration reduced nicotine self-administration in the fixed-ratio phase but increased it in the progressive-ratio phase, reaching statistical significance with the highest dose (1 mg/kg, i.p.). Likewise, relapse induced by both nicotine readministration and the presentation of nicotine-conditioned stimuli was markedly enhanced by the administration of WIN55,212-2. In a complementary manner, and to assess whether CB1r, CB2r, or both mediated the effects observed in the relapse phase, pretreatment with selective antagonists of both receptors (rimonabant (1 mg/kg, i.p.) and AM630 (5 mg/kg, i.p.), respectively) was performed. Although CB1r blockade succeeded in abrogating the relapse of the animals induced by nicotine or conditioned stimuli, no changes occurred when the CB2r antagonist was administered. Thus, it was concluded that CB2r would not be involved, diverting attention to CB1r. The same authors published another study in 2012 [89] to evaluate whether CB2r could play any relevant role in modulating the reinforcing motivational effects of nicotine. To do so, they again employed an intravenous nicotine self-administration paradigm, analyzing the effects of administration of a CB2r agonist (AM1241, 1 to 10 mg/kg, i.p.) and an antagonist (AM630, 1.25 to 5 mg/kg, i.p.). The results obtained were consistent with those previously published since neither activation nor blockade of CB2r modified nicotine self-administration (fixed and progressive ratio). Likewise, neither did relapse induced by re-exposure to the drug or drug-conditioned stimuli vary. Therefore, the authors reaffirmed their previous conclusion, suggesting that their results did not support the involvement of CB2r in self-administration or nicotine-seeking behavior. However, a year later, our group published a study that unequivocally supported the active involvement of CB2r in the regulation of nicotine’s reinforcing properties [49]. Different experimental paradigms were employed to assess reinforcement (CPP), motivation (intravenous self-administration of nicotine), and withdrawal (assessment of somatic signs) induced by nicotine. A genetic approach was employed to analyze the role of CB2r, using CB2-/- mice and their respective WT mice, and a pharmacological approach was performed by administering the CB2r antagonist AM630 to WT mice. The results showed that nicotine could not induce conditioning place preference in CB2-/- mice at any of the doses tested (0.5, 0.7, and 1 mg/kg, i.p.). Likewise, AM630 (1 mg/kg, i.p.) blocked nicotine-mediated effects. On the other hand, CB2-/- mice hardly self-administered nicotine, and their level of motivation in the progressive ratio phase was much lower than WT mice. Furthermore, administration of AM630 (1 and 3 mg/kg, i.p.) reduced intravenous self-administration in both fixed and progressive ratio phases. Finally, and concerning the evaluation of the nicotine withdrawal syndrome precipitated by the administration of a specific nicotinic antagonist (mecamylamine), the results again showed that CB2-/- mice hardly presented the characteristic somatic signs and that these were significantly and dose-dependently reduced by AM630 (1 and 3 mg/kg, i.p.).

**Figure 3:**
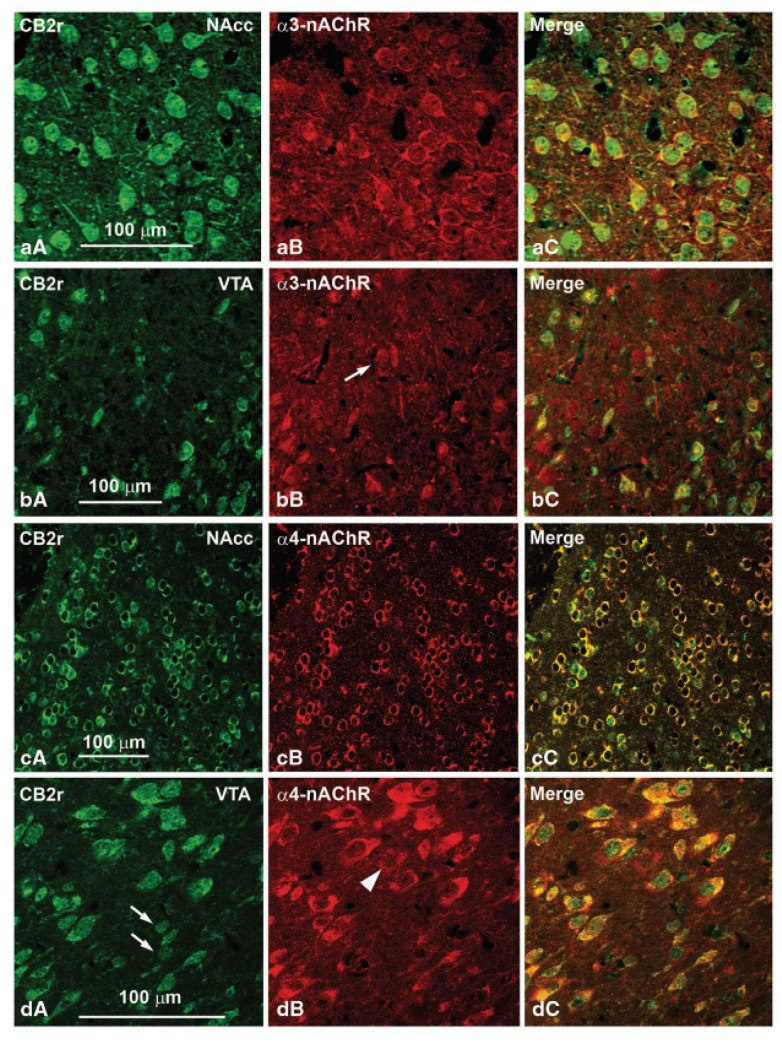
Confocal photomicrographs showing immunolabeling for CB2 receptors (CB2r) and α3 and α4 subunits of the cholinergic nicotinic receptor (α3-nAChR and α4-nAChR) in the ventral tegmental area (VTA) and the nucleus accumbens (NAcc) of wild-type (WT) mice. Double labeling (yellow cells in panels aC, bC, cC and dC) indicates that α3- and α4-nAChRs colocalize with CB2r immunoreactive cells. Image adapted from Navarrete et al. Neuropsychopharmacology (2013) 38(12), 2515–2524.

The brain changes related to the behavioral effects derived from the genetic deletion of CNR2 were evaluated by real-time PCR in CB2-/- and WT mice. Gene expression of WT and α3 and α4 subunits of the nicotinic cholinergic receptor were analyzed in the VTA. The results indicated that gene expression of both TH and nicotinic subunits was significantly reduced in CB2-/- mice. Moreover, the possible colocalization of CB2r with these subunits in the VTA and NAcc of WT mice was evaluated. The results revealed colocalization of CB2r with the α3 and α4 subunits of the nicotinic cholinergic receptor in areas of the reward system (Figure 3). This finding could suggest the functional cooperation of both receptors and their involvement in regulating nicotine effects at the mesolimbic level. Shortly after that, another paper was published [82], confirming that CB2r regulated nicotine-induced conditioning place preference effects. Consistent with our results [49], both genetic deletion (CB2-/- mice) and pharmacological blockade (administration of the CB2r antagonist SR144528) completely abolished nicotine-induced place preference.

Furthermore, it is noteworthy that administering a CB2r agonist (O-1966) and a subeffective dose of nicotine increased the stay in the drug-conditioned compartment. However, in this case, CB2-/- mice showed a similar response profile to WT mice when nicotine withdrawal syndrome was pharmacologically precipitated by mecamylamine administration. Therefore, the authors concluded that CB2r is necessary to induce nicotine conditioning preference, apparently not regulating withdrawal-associated symptomatology. The discrepancies between the two studies may be due to different experimental circumstances, such as using another mouse strain (C57BL/6J vs. Swiss ICR) or the different duration of nicotine administration before the precipitation of withdrawal syndrome with mecamylamine (7 days vs. 14 days).

Recently, Canseco-Alba et al. [86] reported the absence of nicotine-induced place preference conditioning in DAT-Cnr2-/- mice, suggesting an effect that reproduces the behavior shown by CB2-/- mice [49,82]. Similarly, pretreatment with the agonist JWH133 (3 mg/kg, i.p.) blocked such conditioning in WT mice. This effect was similar to that obtained after administration of the antagonist AM630 (3 mg/kg, i.p.) [49], probably because of differences in the timing and pattern of administration (repeatedly during the conditioning sessions or punctually in the postconditioning session).

In summary, the work carried out in recent years has allowed us to deepen our understanding of the relationship between the different phases of nicotine dependence and CB2r (Table 3), pointing to dopaminergic transmission as a possible interaction pathway. However, further studies are needed to explore whether CB2r could be helpful as a new pharmacological target in the therapeutic approach to nicotine dependence.

## 5. Discussion

The results compiled in this article suggest the functional involvement of CB2r in the effects produced by drugs of abuse such as alcohol, cocaine, or nicotine at the CNS level. First, the discovery of the presence of CB2r in brain areas that are part of the reward system, its colocalization with receptors involved in mediating the reinforcing and motivational effects of different substances of abuse, and the findings on its involvement in the regulation of dopamine release at the mesolimbic level, support the relevance of CB2r in the field of addiction. Secondly, the experimental approaches carried out at the preclinical level employing genetic and/or pharmacological manipulation of CB2r allowed us to understand the role played by this receptor in the regulation of different behavioral traits related to the addictive process such as reinforcement, motivation, relapse, psychomotor effects or withdrawal symptoms.

Despite the advances made in recent years, mainly through animal models, further studies are still needed to improve our knowledge about the involvement of CB2r in SUD. From a pharmacological perspective, designs that assess more comprehensive dose ranges and more prolonged treatment durations are required to analyze CB2r activation or blockade effects in the longer term. Likewise, behavioral procedures that specifically evaluate traits associated with relapse, abstinence, or psychomotor sensitization will be advantageous due to their greater translational power towards clinical problems. On the other hand, it will be essential to elucidate the neurobiological mechanisms underlying the effects mediated by CB2r, being necessary to apply multidisciplinary experimental approaches that help to understand better the processes involved. According to the previously mentioned findings, the functional regulation of dopaminergic neurotransmission in brain areas of the reward system such as the VTA or the NAcc would play an important role.

In addition to preclinical studies, it is essential to analyze samples from patients diagnosed with SUD. The results obtained from the analysis of biological samples (blood, cerebrospinal fluid) or postmortem brain samples, and from the information provided by studies using neuroimaging techniques or evaluating different psychophysiological aspects, will be unquestionably valuable to improve our knowledge of the role of CB2r in the addictive process. Likewise, advances in preclinical approaches could lead to clinical trials to assess the safety and tolerability of compounds acting at the level of CB2r and finally analyze their efficacy. In this regard, it should be noted that there are currently precedents for the administration in patients of a compound with CB2r agonist properties (GW842166X; reference of the clinical trial published on the clinicaltrials.gov website: NCT00511524), a fact that partly paves the way for the future development of related molecules that could be useful in the treatment of SUD. It is also important to note that currently available CB2r agonist and antagonist compounds can also act on CB1r depending on the administered dose. This is a limitation given the possibility of characteristic adverse effects (reinforcing properties, cognitive alterations, psychotic disorders). Therefore, the synthesis of substances that act as allosteric modulators may be of great utility rather than agonists or antagonists. This approach would confer advantages such as better pharmacological selectivity and functional modulation dependent on the presence of endocannabinoids in specific brain regions [90].

In conclusion, there is a growing interest in exploring the therapeutic potential of strategies to modulate CB2r-mediated effects in treating addictive disorders. In this sense, the future development of new pharmacological tools focused on CB2r may help to have more specific approaches that, alone or in combination with the treatments already known, increase the therapeutic success in the management of SUD.

## Figures and Tables

**Table 1:** Main findings supporting the involvement of CB2r in the regulation of the reinforcing and motivational effects of alcohol.

CB2r & Alcohol-ANIMAL STUDIES				
Genetic Manipulation CB2r				
Genetic manipulation	Specie	Experimental paradigm	Results	References
CB2r deletion, knock-out (CB2-/- and CNR2 KO)	Swiss ICR and C57BL/6J mice	CPP	↑ Conditioned place preference	[50]
		VC	↑ Voluntary ethanol consumption and preference	
		OESA	↑ Motivation to drink ethanol	
CB2r deletion, knock-out (CNR2 KO)	C57BL/6J mice	FD IFD	No differences	[72]
		VC	↑ Alcohol drinking in group-housing conditions	
CB2r deletion, knock-out (CNR2 KO)	C57BL/6J mice	CPP	↑ Conditioned place preference	[73]
CB2r deletion, in dopaminergic neurons, knock out condicional (DAT-Cnr2-/-)	C57BL/6J mice	CPP	↓ Conditioned place preference	[77]
		VC		
**Pharmacological manipulation of CB2r**				
**Doses**	**Specie**	**Experimental paradigm**	**Results**	**References**
β-caryophyllene, CB2r agonist (25, 50 and 100 mg/kg)	C57BL/6J mice	CPP	↓ Conditioned place preference	[74]
		VC	↓ Voluntary ethanol consumption and preference	
JWH133, CB2r agonist (10 y 20 mg/kg)	HS/Ibg mice	CPP	No differences	[73]
		VC	No differences	
JWH133, CB2r agonist (5 mg/kg)	C57BL/6J mice	CPP	↓ Conditioned place preference	[28]
JWH133, CB2r agonist (1 mg/kg)	C57BL/6J mice	OESA	↓ Motivation to drink ethanol	[75]
**CB2r & Alcohol-Human Studies**				
**Diagnosis**	**Population**	**Variable**	**Results**	**References**
SUD	Japanese	Q63R (CB2r) polymorphism association-SUD	↑ Incidence	[51,68]
SUD	Caucasian	Relative gene expression (CB2r)	↓ DLPFCx y NAcc	[71]

↑: increase, ↓: decrease, CPP: Conditioned Place Preference, VC: Voluntary Consumption, ESA: Oral Ethanol Self-Administration, FD: Forced Drinking, IFD: Intermittent Forced Drinking, SUD: Substance Use Disorder, DLPFCx: Dorsolateral Prefrontal Cortex, NAcc: Nucleus Accumbens.

**Table 2:** Main findings supporting the involvement of CB2r in the regulation of the reinforcing and motivational effects of cocaine.

CB2r & Cocaine-Animals Studies				
**Genetic Manipulation CB2r**				
**Genetic manipulation**	**Specie**	**Experimental paradigm**	**Results**	**References**
Overexpression of the CB2r (CB_2_xP)	Swiss ICR mice	SM	↓ Cocaine-induced motor sensitization	[48]
		CPP	↓ Conditioned place preference	
		ISA	↓ Motivation for cocaine consumption	
CB2r deletion in dopaminergic neurons, conditional knock-out (DAT-Cnr2-/-)	C57BL/6J mice	MA	↑ Cocaine-induced hyperactivity	[86]
		MS	↑ Cocaine-induced motor sensitization (10 mg/kg)	
		CPP	↓ Cocaine-induced motor sensitization (20 mg/kg) ↑ Conditioned place preference	
**Pharmacological manipulation of CB2r**				
**Doses**	**Specie**	**Experimental paradigm**	**Results**	**References**
JWH133, CB2r antagonist (10 and 20 mg/kg)	C57BL/6J mice	ISA	↓ Motivation for cocaine consumption	[60]
		MA	↓ Cocaine-induced locomotor effect	
GW405833, CB2r agonist (3 and 10 mg/kg)	C57BL/6J mice	ISA	↓ Motivation for cocaine consumption	[81]
SR144528, CB2r antagonist (0.1, 0.3 and 1 mg/kg)	Wistar rats	ISA	No differences in motivation for cocaine consumption ↓ Cocaine-triggered relapse (not conditioned stimulus-triggered relapse)	
O-1966, CB2r antagonist (1, 3, 5, 10 and 20 mg/kg)	C57BL/6J mice	CPP	↓ Conditioned place preference	[82]
JWH133, CB2r agonist (10 and 20 mg/kg)	C57BL/6J mice	ISA	↓ Motivation for cocaine consumption	[83]
	Long-Evans rats	ISA	↑ Motivation for cocaine consumption	
JWH133, CB2r agonist (3 mg/kg)	C57BL/6J mice	CPP	↓ Conditioned place preference	[86]

↑: increase,↓: decrease, MS: Motor Sensitization, CPP: Conditioned Place Preference, ISA: Intravenous Self-Administration, MA: Motor Activity.

**Table 3:** Main findings supporting the involvement of CB2r in the regulation of the reinforcing and motivational effects of nicotine.

CB2r & Nicotine-Animal studies				
**Genetic manipulation CB2r**				
**Genetic manipulation**	**Specie**	**Experimental paradigm**	**Results**	**References**
CB2r deletion, *knock-out* (CB2-/-)	Swiss ICR mice	CPP	↓ Conditioned place preference	[49]
		ISA	↓ Motivation for nicotine consumption	
		WS	↓ Withdrawal signs	
CB2r deletion, *knock-out* (CB2-/-)	C57BL/6J mice	CPP	↓ Conditioned place preference	[82]
CB2r deletion in dopaminergic neurons, conditional knock-out (DAT-Cnr2-/-)	C57BL/6J mice	CPP	↓ Conditioned place preference	[86]
**Pharmacological manipulation of CB2r**				
**Doses**	**Specie**	**Experimental paradigm**	**Results**	**References**
SR144528, CB2r agonist (3 mg/kg)	Wistar Rats	DT	No differences	[87]
WIN55,212-2, CB1r/CB2r agonist (1 mg/kg) + Rimonabant, CB1r agonist (1 mg/kg) or AM630, CB2r antagonist (5 mg/kg)	Long-Evans rats	ISA	↑ WIN55,212-2-induced nicotine relapse, blocked by rimonabant but not by AM630	[88]
AM1241, CB2r agonist (1–10 mg/kg)	Long-Evans rats	ISA	No differences	[89]
AM630, CB2r agonist (1.25–5 mg/kg)	Long-Evans rats	ISA	No differences	
AM630, CB2r antagonist (1 mg/kg)	Swiss ICR mice	CPP	↓ Conditioned place preference	[49]
		ISA	↓ Motivation for nicotine consumption	
		WA	↓ Withdrawal signs	
SR144528, CB2r antagonist (3 mg/kg) O-1966, CB2r agonist (1–20 mg/kg)	C57BL/6J mice	CPP	↓ Conditioned place preference	[82]
	C57BL/6J mice	CPP	↑ Conditioned place preference	

DT: Discrimination Task, ISA: Intravenous Self-Administration, WS: Withdrawal Syndrome, CPP: Conditioned Place Preference, ↑: increase, ↓: decrease.

## References

[1] APA (2014). Diagnostic and Statistical Manual of Mental Disorders.

[2] United Nations Office on Drugs and Crime (2021). World Drug Report.

[3] WHO (2018). Global Status Report on Alcohol and Health.

[4] Piomelli D. (2003). The molecular logic of endocannabinoid signalling. Nat. Rev. Neurosci..

[5] Zou S., Kumar U. (2018). Cannabinoid Receptors and the Endocannabinoid System: Signaling and Function in the Central Nervous System. Int. J. Mol. Sci..

[6] Katona I., Freund T.F. (2012). Multiple Functions of Endocannabinoid Signaling in the Brain. Annu. Rev. Neurosci..

[7] Mackie K. (2005). Distribution of cannabinoid receptors in the central and peripheral nervous system. Cannabinoids. Handbook of Experimental Pharmacology.

[8] De Fonseca F.R., Del Arco I., Bermudez-Silva F.J., Bilbao A., Cippitelli A., Navarro M. (2005). The Endocannabinoid System: Physiology and Pharmacology. Alcohol Alcohol..

[9] Tsou K., Brown S., Sañudo-Peña M., Mackie K., Walker J. (1998). Immunohistochemical distribution of cannabinoid CB1 receptors in the rat central nervous system. Neuroscience.

[10] Matsuda L.A., Lolait S.J., Brownstein M.J., Young A.C., Bonner T.I. (1990). Structure of a cannabinoid receptor and functional expression of the cloned cDNA. Nature.

[11] Gutiérrez-Rodríguez A., Puente N., Elezgarai I., Ruehle S., Lutz B., Reguero L., Gerrikagoitia I., Marsicano G., Grandes P. (2017). Anatomical characterization of the cannabinoid CB1receptor in cell-type-specific mutant mouse rescue models. J. Comp. Neurol..

[12] Piazza P.V., Cota D., Marsicano G. (2017). The CB1 Receptor as the Cornerstone of Exostasis. Neuron.

[13] Busquets-Garcia A., Bains J., Marsicano G. (2018). CB1 Receptor Signaling in the Brain: Extracting Specificity from Ubiquity. Neuropsychopharmacology.

[14] Munro S., Thomas K.L., Abu-Shaar M. (1993). Molecular characterization of a peripheral receptor for cannabinoids. Nature.

[15] Galiegue S., Mary S., Marchand J., Dussossoy D., Carriere D., Carayon P., Bouaboula M., Shire D., Le Fur G., Casellas P. (1995). Expression of Central and Peripheral Cannabinoid Receptors in Human Immune Tissues and Leukocyte Subpopulations. Eur. J. Biochem..

[16] Cabral G.A., Griffin-Thomas L. (2009). Emerging role of the cannabinoid receptor CB2in immune regulation: Therapeutic prospects for neuroinflammation. Expert Rev. Mol. Med..

[17] Benito C., Nunez E., Tolon R.M., Carrier E.J., Rabano A., Hillard C.J., Romero J. (2003). Cannabinoid CB2 receptors and fatty acid amide hydrolase are selectively overexpressed in neuritic plaque-associated glia in Alzheimer’s disease brains. J. Neurosci..

[18] Yiangou Y., Facer P., Durrenberger P., Chessell I.P., Naylor A., Bountra C., Banati R.R., Anand P. (2006). COX-2, CB2 and P2X7-immunoreactivities are increased in activated microglial cells/macrophages of multiple sclerosis and amyotrophic lateral sclerosis spinal cord. BMC Neurol..

[19] Guzmán M., Sanchez C., Galve-Roperh I. (2000). Control of the cell survival/death decision by cannabinoids. J. Mol. Med..

[20] Van Sickle M.D., Duncan M., Kingsley P.J., Mouihate A., Urbani P., Mackie K., Stella N., Makriyannis A., Piomelli D., Davison J.S. (2005). Identification and Functional Characterization of Brainstem Cannabinoid CB 2 Receptors. Science.

[21] Gong J.-P., Onaivi E.S., Ishiguro H., Liu Q.-R., Tagliaferro P.A., Brusco A., Uhl G.R. (2006). Cannabinoid CB2 receptors: Immunohistochemical localization in rat brain. Brain Res..

[22] Onaivi E.S. (2006). Neuropsychobiological Evidence for the Functional Presence and Expression of Cannabinoid CB2 Receptors in the Brain. Neuropsychobiology.

[23] Onaivi E.S., Ishiguro H., Gong J., Patel S., Perchuk A., Meozzi P.A., Myers L., Mora Z., Tagliaferro P., Gardner E. (2006). Discovery of the Presence and Functional Expression of Cannabinoid CB2 Receptors in Brain. Ann. N. Y. Acad. Sci..

[24] García-Gutiérrez M., Pérez-Ortiz J., Gutiérrez-Adán A., Manzanares J. (2010). Depression-resistant endophenotype in mice overexpressing cannabinoid CB2 receptors. Br. J. Pharmacol..

[25] Zhang H., Gao M., Liu Q.-R., Bi G.-H., Li X., Yang H.-J., Gardner E.L., Wu J., Xi Z.-X. (2014). Cannabinoid CB2receptors modulate midbrain dopamine neuronal activity and dopamine-related behavior in mice. Proc. Natl. Acad. Sci. USA.

[26] Cabral G.A., Raborn E.S., Griffin L., Dennis J., Marciano-Cabral F. (2008). CB2 receptors in the brain: Role in central immune function. Br. J. Pharmacol..

[27] García-Gutiérrez M.S., Navarrete F., Navarro G., Reyes-Resina I., Franco R., Lanciego J.L., Giner S., Manzanares J. (2018). Alterations in Gene and Protein Expression of Cannabinoid CB2 and GPR55 Receptors in the Dorsolateral Prefrontal Cortex of Suicide Victims. Neurotherapeutics.

[28] Liu Q.-R., Canseco-Alba A., Zhang H.-Y., Tagliaferro P., Chung M., Dennis E., Sanabria B., Schanz N., Escosteguy-Neto J.C., Ishiguro H. (2017). Cannabinoid type 2 receptors in dopamine neurons inhibits psychomotor behaviors, alters anxiety, depression and alcohol preference. Sci. Rep..

[29] Navarrete F., García-Gutiérrez M.S., Aracil-Fernández A., Lanciego J.L., Manzanares J. (2018). Cannabinoid CB1 and CB2 Receptors, and Monoacylglycerol Lipase Gene Expression Alterations in the Basal Ganglia of Patients with Parkinson’s Disease. Neurotherapeutics.

[30] Pertwee R.G., Howlett A.C., Abood M.E., Alexander S.P., Di Marzo V., Elphick M.R., Greasley P.J., Hansen H.S., Kunos G., Mackie K. (2010). International Union of Basic and Clinical Pharmacology. LXXIX. Cannabinoid Receptors and Their Ligands: Beyond CB1and CB2. Pharmacol. Rev..

[31] Kano M., Ohno-Shosaku T., Hashimotodani Y., Uchigashima M., Watanabe M. (2009). Endocannabinoid-Mediated Control of Synaptic Transmission. Physiol. Rev..

[32] Alger B.E., Kim J. (2011). Supply and demand for endocannabinoids. Trends Neurosci..

[33] Devane W.A., Hanus L., Breuer A., Pertwee R.G., Stevenson L.A., Griffin G., Gibson D., Mandelbaum A., Etinger A., Mechoulam R. (1992). Isolation and structure of a brain constituent that binds to the cannabinoid receptor. Science.

[34] Mechoulam R., Ben-Shabat S., Hanus L., Ligumsky M., Kaminski N.E., Schatz A.R., Gopher A., Almog S., Martin B.R., Compton D.R. (1995). Identification of an endogenous 2-monoglyceride, present in canine gut, that binds to cannabinoid receptors. Biochem. Pharmacol..

[35] Manzanares J., Cabañero D., Puente N., García-Gutiérrez M.S., Grandes P., Maldonado R. (2018). Role of the endocannabinoid system in drug addiction. Biochem. Pharmacol..

[36] Filip M., Gołda A., Zaniewska M., McCreary A.C., Nowak E., Kolasiewicz W., Przegaliński E. (2007). Involvement of cannabinoid CB1 receptors in drug addiction: Effects of rimonabant on behavioral responses induced by cocaine. Pharmacol. Rep..

[37] Parolaro D., Vigano’ D., Realini N., Rubino T. (2008). Role of endocannabinoids in regulating drug dependence. Neuropsychiatr. Dis. Treat..

[38] Le Foll B., Forget B., Aubin H.-J., Goldberg S.R. (2008). Blocking cannabinoid CB1 receptors for the treatment of nicotine dependence: Insights from pre-clinical and clinical studies. Addict. Biol..

[39] Le Foll B., Goldberg S.R. (2005). Cannabinoid CB1 Receptor Antagonists as Promising New Medications for Drug Dependence. J. Pharmacol. Exp. Ther..

[40] Malinen H., Hyytiä P. (2008). Ethanol Self-Administration Is Regulated by CB1 Receptors in the Nucleus Accumbens and Ventral Tegmental Area in Alcohol-Preferring AA Rats. Alcohol. Clin. Exp. Res..

[41] Lallemand F., De Witte P. (2006). SR147778, a CB1 Cannabinoid Receptor Antagonist, Suppresses Ethanol Preference in Chronically Alcoholized Wistar Rats. Alcohol.

[42] Lallemand F., Soubrié P., De Witte P. (2004). Effects of cb1 cannabinoid receptor blockade on ethanol preference after chronic alcohol administration combined with repeated re-exposures and withdrawals. Alcohol Alcohol..

[43] Lallemand F., Soubrié P.H., De Witte P.H. (2001). Effects of CB1 cannabinoid receptor blockade on ethanol preference after chronic ethanol administration. Alcohol. Clin. Exp. Res..

[44] Soyka M., Koller G., Schmidt P., Lesch O.-M., Leweke M., Fehr C., Gann H., Mann K.F. (2008). Cannabinoid Receptor 1 Blocker Rimonabant (SR 141716) for Treatment of Alcohol Dependence: Results from a placebo-controlled, double-blind trial. J. Clin. Psychopharmacol..

[45] George D.T., Herion D.W., Jones C.L., Phillips M.J., Hersh J., Hill D., Heilig M., Ramchandani V.A., Geyer C., Spero D.E. (2009). Rimonabant (SR141716) has no effect on alcohol self-administration or endocrine measures in nontreatment-seeking heavy alcohol drinkers. Psychopharmacology.

[46] Morales M., Bonci A. (2012). Getting to the core of addiction: Hooking CB2 receptor into drug abuse?. Nat. Med..

[47] Yang P., Wang L., Xie X.-Q. (2012). Latest advances in novel cannabinoid CB2 ligands for drug abuse and their therapeutic potential. Future Med. Chem..

[48] Aracil-Fernández A., Trigo J.M., García-Gutiérrez M.S., Álvaro A.O., Ternianov A., Navarro D., Robledo P., Berbel P., Maldonado R., Manzanares J. (2012). Decreased Cocaine Motor Sensitization and Self-Administration in Mice Overexpressing Cannabinoid CB_2_ Receptors. Neuropsychopharmacology.

[49] Navarrete F., Rodriguez-Arias M., Martin-García E., Navarro D., García-Gutiérrez M.S., Aguilar M.A., Aracil-Fernández A., Berbel P., Miñarro J., Maldonado R. (2013). Role of CB2 Cannabinoid Receptors in the Rewarding, Reinforcing, and Physical Effects of Nicotine. Neuropsychopharmacology.

[50] Ortega-Álvaro A., Ternianov A., Aracil-Fernández A., Navarrete F., García-Gutiérrez M.S., Manzanares J. (2015). Role of cannabinoid CB2receptor in the reinforcing actions of ethanol. Addict. Biol..

[51] Onaivi E.S., Ishiguro H., Gong J.-P., Patel S., Meozzi P.A., Myers L., Perchuk A., Mora Z., Tagliaferro P.A., Gardner E. (2008). Brain Neuronal CB2 Cannabinoid Receptors in Drug Abuse and Depression: From Mice to Human Subjects. PLoS ONE.

[52] Benito C., Tolón R.M., Pazos M.R., Nuñez E., Castillo A.I., Romero J. (2008). Cannabinoid CB2 receptors in human brain inflammation. Br. J. Pharmacol..

[53] Turcotte C., Blanchet M.-R., LaViolette M., Flamand N. (2016). The CB2 receptor and its role as a regulator of inflammation. Cell. Mol. Life Sci..

[54] Fernández-Ruiz J., Pazos M.R., García-Arencibia M., Sagredo O., Ramos J.A. (2008). Role of CB2 receptors in neuroprotective effects of cannabinoids. Mol. Cell. Endocrinol..

[55] Ternianov A., Pérez-Ortiz J.M., Solesio M.E., García-Gutiérrez M.S., Álvaro A.O., Navarrete F., Leiva C., Galindo M.F., Manzanares J. (2012). Overexpression of CB2 cannabinoid receptors results in neuroprotection against behavioral and neurochemical alterations induced by intracaudate administration of 6-hydroxydopamine. Neurobiol. Aging.

[56] García-Gutiérrez M.S., García-Bueno B., Zoppi S., Leza J.C., Manzanares J. (2012). Chronic blockade of cannabinoid CB2 receptors induces anxiolytic-like actions associated with alterations in GABAA receptors. Br. J. Pharmacol..

[57] García-Gutiérrez M.S., Manzanares J. (2011). Overexpression of CB2 cannabinoid receptors decreased vulnerability to anxiety and impaired anxiolytic action of alprazolam in mice. J. Psychopharmacol..

[58] Hu B., Doods H., Treede R.-D., Ceci A. (2009). Depression-like behaviour in rats with mononeuropathy is reduced by the CB2-selective agonist GW405833. Pain.

[59] Liu Q.-R., Pan C.-H., Hishimoto A., Li C.-Y., Xi Z.-X., Llorente-Berzal A., Viveros M.-P., Ishiguro H., Arinami T., Onaivi E.S. (2009). Species differences in cannabinoid receptor 2 (CNR2gene): Identification of novel human and rodent CB2 isoforms, differential tissue expression and regulation by cannabinoid receptor ligands. Genes Brain Behav..

[60] Xi Z.-X., Peng X., Li X., Song R., Zhang H., Liu Q.-R., Yang H.-J., Bi G.-H., Li J., Gardner E.L. (2011). Brain cannabinoid CB2 receptors modulate cocaine’s actions in mice. Nat. Neurosci..

[61] Zhang H.-Y., Xi Z.-X. (2017). Chapter 70—Cannabinoid CB2 receptor: A new target for treatment of cocaine addiction. The Neuroscience of Cocaine Mechanisms and Treatment.

[62] Ma Z., Gao F., Larsen B., Gao M., Luo Z., Chen D., Ma X., Qiu S., Zhou Y., Xie J. (2019). Mechanisms of cannabinoid CB2 receptor-mediated reduction of dopamine neuronal excitability in mouse ventral tegmental area. EBioMedicine.

[63] García-Gutiérrez M.S., Ortega-Álvaro A., Busquets-García A., Pérez-Ortiz J.M., Caltana L., Ricatti M.J., Brusco A., Maldonado R., Manzanares J. (2013). Synaptic plasticity alterations associated with memory impairment induced by deletion of CB2 cannabinoid receptors. Neuropharmacology.

[64] Ratano P., Petrella C., Forti F., Passeri P.P., Morena M., Palmery M., Trezza V., Severini C., Campolongo P. (2018). Pharmacological inhibition of 2-arachidonoilglycerol hydrolysis enhances memory consolidation in rats through CB2 receptor activation and mTOR signaling modulation. Neuropharmacology.

[65] Navarrete F., Pérez-Ortiz J.M., Manzanares J. (2012). Cannabinoid CB2 receptor-mediated regulation of impulsive-like behaviour in DBA/2 mice. Br. J. Pharmacol..

[66] Rodríguez-Arias M., Navarrete F., Blanco-Gandía M.C., Arenas M.C., Aguilar M.A., Bartoll-Andrés A., Valverde O., Miñarro J., Manzanares J. (2015). Role of CB2 receptors in social and aggressive behavior in male mice. Psychopharmacology.

[67] Marco E.M., García-Gutiérrez M.S., Bermúdez-Silva F.-J., Moreira F., Guimarães F., Manzanares J., Viveros M.-P. (2011). Endocannabinoid system and psychiatry: In search of a neurobiological basis for detrimental and potential therapeutic effects. Front. Behav. Neurosci..

[68] Ishiguro H., Iwasaki S., Teasenfitz L., Higuchi S., Horiuchi Y., Saito T., Arinami T., Onaivi E.S. (2006). Involvement of cannabinoid CB2 receptor in alcohol preference in mice and alcoholism in humans. Pharmacogenom. J..

[69] Serrano A., Rivera P., Pavon F.J., Decara J., Suárez J., de Fonseca F.R., Parsons L.H. (2012). Differential Effects of Single versus Repeated Alcohol Withdrawal on the Expression of Endocannabinoid System-Related Genes in the Rat Amygdala. Alcohol. Clin. Exp. Res..

[70] Marín L.S., Pavon F.J., Decara J., Suarez J., Gavito A., Castilla-Ortega E., De Fonseca F.R., Serrano A. (2017). Effects of Intermittent Alcohol Exposure on Emotion and Cognition: A Potential Role for the Endogenous Cannabinoid System and Neuroinflammation. Front. Behav. Neurosci..

[71] Garcia-Gutierrez M.S., Aracil-Fernandez A., Navarrete F., Manzanares J. Cannabinoid CB2 receptor gene expression alterations in the dorsolateral prefrontal cortex and nucleus accumbens of alcoholic patients. Proceedings of the 3rd International Congress on Dual Pathology.

[72] Pradier B., Erxlebe E., Markert A., Rácz I. (2015). Interaction of cannabinoid receptor 2 and social environment modulates chronic alcohol consumption. Behav. Brain Res..

[73] Powers M.S., Breit K.R., Chester J.A. (2015). Genetic Versus Pharmacological Assessment of the Role of Cannabinoid Type 2 Receptors in Alcohol Reward-Related Behaviors. Alcohol. Clin. Exp. Res..

[74] Al Mansouri S., Ojha S., Al Maamari E., Al Ameri M., Nurulain S.M., Bahi A. (2014). The cannabinoid receptor 2 agonist, β-caryophyllene, reduced voluntary alcohol intake and attenuated ethanol-induced place preference and sensitivity in mice. Pharmacol. Biochem. Behav..

[75] Navarrete F., García-Gutiérrez M.S., Manzanares J. (2018). Pharmacological regulation of cannabinoid CB2 receptor modulates the reinforcing and motivational actions of ethanol. Biochem. Pharmacol..

[76] Martín-Sánchez A., Warnault V., Montagud-Romero S., Pastor A., Mondragón N., De La Torre R., Valverde O. (2019). Alcohol-induced conditioned place preference is modulated by CB2 cannabinoid receptors and modifies levels of endocannabinoids in the mesocorticolimbic system. Pharmacol. Biochem. Behav..

[77] Canseco-Alba A., Schanz N., Ishiguro H., Liu Q.-R., Onaivi E.S. (2018). Behavioral Evaluation of Seeking and Preference of Alcohol in Mice Subjected to Stress. Bio-Protocol.

[78] Trebicka J., Racz I., Siegmund S.V., Cara E., Granzow M., Schierwagen R., Klein S., Wojtalla A., Hennenberg M., Huss S. (2011). Role of cannabinoid receptors in alcoholic hepatic injury: Steatosis and fibrogenesis are increased in CB2 receptor-deficient mice and decreased in CB1 receptor knockouts. Liver Int..

[79] Denaës T., Lodder J., Chobert M.-N., Ruiz I., Pawlotsky J.-M., Lotersztajn S., Teixeira-Clerc F. (2016). The Cannabinoid Receptor 2 Protects Against Alcoholic Liver Disease via a Macrophage Autophagy-Dependent Pathway. Sci. Rep..

[80] Rivera P., Blanco E., Bindila L., Alen F., Vargas A., Rubio L., Pavon F.J., Serrano A., Lutz B., De Fonseca F.R. (2015). Pharmacological activation of CB2 receptors counteracts the deleterious effect of ethanol on cell proliferation in the main neurogenic zones of the adult rat brain. Front. Cell. Neurosci..

[81] Adamczyk P., Miszkiel J., McCreary A.C., Filip M., Papp M., Przegaliński E. (2012). The effects of cannabinoid CB1, CB2 and vanilloid TRPV1 receptor antagonists on cocaine addictive behavior in rats. Brain Res..

[82] Ignatowska-Jankowska B.M., Muldoon P.P., Lichtman A.H., Damaj M.I. (2013). The cannabinoid CB2 receptor is necessary for nicotine-conditioned place preference, but not other behavioral effects of nicotine in mice. Psychopharmacology.

[83] Zhang H., Bi G.-H., Li X., Li J., Qu H., Zhang S.-J., Li C.-Y., Onaivi E.S., Gardner E.L., Xi Z.-X. (2015). Species Differences in Cannabinoid Receptor 2 and Receptor Responses to Cocaine Self-Administration in Mice and Rats. Neuropsychopharmacology.

[84] García-Cabrerizo R., García-Fuster M.J. (2016). Opposite regulation of cannabinoid CB1 and CB2 receptors in the prefrontal cortex of rats treated with cocaine during adolescence. Neurosci. Lett..

[85] Bystrowska B., Frankowska M., Smaga I., Pomierny-Chamiolo L., Filip M. (2018). Effects of Cocaine Self-Administration and Its Extinction on the Rat Brain Cannabinoid CB1 and CB2 Receptors. Neurotox. Res..

[86] Canseco-Alba A., Schanz N., Sanabria B., Zhao J., Lin Z., Liu Q.-R., Onaivi E.S. (2019). Behavioral effects of psychostimulants in mutant mice with cell-type specific deletion of CB2 cannabinoid receptors in dopamine neurons. Behav. Brain Res..

[87] Zaniewska M., McCreary A.C., Przegaliński E., Filip M. (2006). Evaluation of the role of nicotinic acetylcholine receptor subtypes and cannabinoid system in the discriminative stimulus effects of nicotine in rats. Eur. J. Pharmacol..

[88] Gamaleddin I., Wertheim C., Zhu A.Z., Coen K.M., Vemuri K., Makryannis A., Goldberg S.R., Le Foll B. (2012). Cannabinoid receptor stimulation increases motivation for nicotine and nicotine seeking. Addict. Biol..

[89] Gamaleddin I., Zvonok A., Makriyannis A., Goldberg S.R., Le Foll B. (2012). Effects of a Selective Cannabinoid CB2 Agonist and Antagonist on Intravenous Nicotine Self Administration and Reinstatement of Nicotine Seeking. PLoS ONE.

[90] Gado F., Di Cesare Mannelli L., Lucarini E., Bertini S., Cappelli E., Digiacomo M., Stevenson L.A., Macchia M., Tuccinardi T., Ghelardini C. (2018). Identification of the First Synthetic Allosteric Modulator of the CB2 Receptors and Evidence of Its Efficacy for Neuropathic Pain Relief. J. Med. Chem..

